# Safety and efficacy of supraciliary dexamethasone implantation for macular oedema: a preliminary comparative study

**DOI:** 10.1038/s41433-024-03570-8

**Published:** 2025-01-07

**Authors:** Selim Doganay, Gamze Ucan Gunduz, Mehmet Omer Kiristioglu, Elif Demirel, Ozgur Yalcinbayir

**Affiliations:** Department of Ophthalmology, Bursa Uludag University School of Medicine, Bursa, Turkey

**Keywords:** Retinal diseases, Outcomes research

## Abstract

**Purpose:**

To evaluate the efficacy and safety of dexamethasone implantation in the supraciliary (SC) space, a novel and potential effective implantation site, compared to intravitreal (IV) application.

**Methods:**

This prospective study included 39 eyes of 38 patients with macular oedema (ME) who underwent SC and IV dexamethasone implantation (SC-DEX and IV-DEX). Patients were randomly assigned to treatment groups and followed for 3 months. Preoperative and postoperative assessments included maximum retinal thickness (MRT), change in central retinal thickness between consecutive visits (ΔCRT), intraocular pressure (IOP), and best corrected visual acuity (BCVA).

**Results:**

Both SC-DEX and IV-DEX groups showed significant MRT reductions during at follow-up. In the SC group, MRT significantly decreased at 1st and 3rd months (*p* = 0.0002 for both), but not at 1st week (*p* = 0.2517). In the IV-DEX group, significant reductions in MRT were observed at all postoperative visits: 1st week (*p* = 0.0002), 1st month (*p* = 0.0004), and 3rd month (*p* = 0.0003). There were no significant differences in the change in ΔCRT between the SC-DEX and IV-DEX groups at any visit (*p* > 0.05). IOP did not show significant changes (*p* > 0.05). BCVA improved significantly in the SC group compared to the IV-DEX group during the first week (*p* = 0.014). No other perioperative or postoperative sight-threatening complications were noted in either group, including hypotony or endophthalmitis.

**Conclusion:**

SC-DEX shows promise as an alternative for managing ME, offering similar effectiveness to IV-DEX with safe profile. Further studies are needed to confirm its long-term safety and efficacy.

## Introduction

Corticosteroids are highly potent and effective anti-inflammatory agents. Their anti-inflammatory and anti-neovascularisation effects have established corticosteroids as a common and integral component of ophthalmology practice for treating both anterior and posterior segment diseases [1, 2]. These agents can be administered topically, intraocularly, or periocularly and have become particularly valuable in non-infectious uveitis, macular oedema following retinal vein occlusions or intraocular surgery, and diabetic macular oedema [2–4]. However, the limited efficacy and potential side effects associated with established routes of corticosteroid administration can restrict their use and impede the full realisation of their therapeutic potential.

The supraciliary (anterior suprachoroidal) and suprachoroidal spaces constitute a continuous expandable space. The supraciliary space (SC), an anterior route for aqueous humour outflow, is situated between the ciliary body and the sclera and extends posteriorly into the suprachoroidal space beyond the pars plana [5]. Suprachoroidal and intravitreal steroid applications have the advantage to bypass ocular barriers and compartmentalise the drug around the targeted tissues, resulting in increased efficacy and reduced systemic and local side effects [6–8].

The promising potential of supraciliary and suprachoroidal spaces has generated considerable interest and stimulated further research [9–12]. Recently, the Food and Drug Administration (FDA) approved suprachoroidal triamcinolone acetonide (TA) injections in 2021 [13]. However, the side effect profile of TA remains a significant concern that needs to be addressed.

The dexamethasone (DEX) implant is a biodegradable copolymer designed to deliver corticosteroid gradually and is reported to have fewer side effects compared to TA [14]. Theoretically, SC administration of the dexamethasone implant may offer an alternative to suprachoroidal TA injections.

To date, no studies have investigated the use of the DEX implant in the SC space. This study compares the effectiveness and safety of administering the DEX implant in the SC space with intravitreal application. Herein, we aim to evaluate whether the SC approach is a viable alternative to the intravitreal route for DEX implant injections. Additionally, based on these preliminary results, we intend to discuss the feasibility of the SC route in cases where intravitreal DEX applications are restricted or where TA application is avoided.

## Materials and methods

This university-based prospective and cross-sectional study examined the clinical records of consecutive patients who received one or more injections of 0.7 mg dexamethasone (DEX) implants between January 2023 and August 2023.

Patients aged 18–80 years who were scheduled to receive dexamethasone (DEX) implant injections for various diagnoses were included in this study. Inclusion criteria required a minimum follow-up of 3 months and the acquisition of high-quality anterior and posterior segment optical coherence tomography (OCT) images with a signal strength greater than 20 dB. Cases with ocular diseases (e.g., glaucoma, scleritis), recent ocular surgery within the past 3 months, and IOP measurements greater than 21 mmHg were excluded from the study. Patients who underwent any form of laser photocoagulation in the past 3 months were not included. Additionally, cases with a history of confounding injections, including anti-vascular endothelial growth factor injections in the past month, dexamethasone (DEX) implant injections in the past 6 months, or other types of steroid injections (intravitreal, suprachoroidal, supraciliary, subtenon, or peribulbar) in the past 6 months, were excluded. Due to national health regulations, treatment-naïve patients were not included to the study. Finally, individuals with best-corrected visual acuity (BCVA) of 20/200 or worse in the untreated fellow eye were also excluded.

In this study, patients were randomly assigned to receive either supraciliary (SC-DEX) or intravitreal (IV-DEX) injections. Baseline demographics and treatment indications were recorded before the injections, and details of the injection site (supraciliary [SC-DEX] or intravitreal [IV-DEX]) along with follow-up information were noted by 2 physicians (EK, OY). Comprehensive ophthalmological examinations, including biomicroscopy, fundoscopy, anterior and posterior OCT evaluations, and intraocular pressure (IOP) measurements, were performed by physicians (GUG, MOK) who were blinded to the type of injection administered. These examinations were conducted before the injections and at follow-up visits at 1st week, 1st month, and 3rd month post-injection. BCVA was assessed using the Early Treatment of Diabetic Retinopathy Study (ETDRS) chart, and IOP was measured with Goldmann applanation tonometry, with results adjusted based on pachymetric measurements.

Spectral-domain OCT scans of the retina and supraciliary space were performed using the Spectralis OCT (Heidelberg Engineering GmbH; Heidelberg, Germany). To verify the correct placement of the DEX implant within the supraciliary space, OCT images of the anterior sclera, encompassing the implantation site, were acquired using enhanced depth imaging mode. Reference points were used at baseline and follow-up retinal OCT acquisitions. Spectralis OCT was configured to perform volume scans with dimensions of 30° × 25°, consisting of 32 lines with 512 A-scans per line for macular cube measurements. ETDRS subfield measurements were obtained using the already built-in Spectralis software. The algorithm utilised the average macular thickness measurements from 4 macular quadrants and 3 concentric circles with diameters of 1 mm, 3 mm, and 6 mm [13]. The maximum retinal thickness (MRT) was defined as the highest value among these quadrants. Central retinal thickness changes (ΔCRT) were assessed within the central fovea subfield of the 1-mm diameter circle. ΔCRT was calculated by subtracting the postoperative central retinal thickness from the preoperative central retinal thickness [ΔCRT = (preoperative CRT) − (postoperative CRT)].

A standard injection procedure was followed in all cases, except for differences in scleral penetration between SC-DEX and IV-DEX injections. The commercially available DEX implant (Ozurdex® 0.7 mg, Allergan Inc., Irvine, CA, USA) was used in all cases of this study, with the original needle. All injections were performed by a single right-handed surgeon (SD) using a sterile drape and an operating microscope. Injections were administered 3.5 mm from the limbus in the temporal superior oblique quadrant for the right eye and in the nasal superior oblique quadrant for the left eye, using an eyelid speculum and a forceps. Subconjunctival lidocaine was applied before the injections, and gentle massage was performed to minimise subconjunctival swelling.

In cases with SC-DEX injections, the eye was stabilised at the limbus, and the DEX implant injector was inserted at a 15-degree angle to the sclera. The bevel-up position was maintained while entering the sclera from the 10 o’clock quadrant. Entry was made 3.5 mm from the limbus, with the needle shaft kept parallel to the limbus. The injector was advanced 2–3 mm into the sclera until its metal part was fully within the intrascleral tunnel, allowing for gradual penetration of the sclera. Upon entering the SC space, the button of the injector was depressed slowly to minimise the impact velocity and penetration force of the implant. The injector was then withdrawn, and gentle pressure was applied to the injection site (see Supplementary Material 1). In the IV-DEX injection technique, the bevelled needle was inserted into the sclera at a 45-degree angle relative to the limbus, creating a 1–2 mm full-thickness tunnel through the sclera and pars plana, through which the implant was subsequently injected into the vitreous. Gentle pressure was again applied to the injection site upon withdrawal of the needle.

In this study, patients were instructed to immediately report any changes in vision, eye pain, or conjunctival hyperaemia. Side effects of the drug and injection-related complications within the follow-up period were documented. This study adhered to the tenets of the Declaration of Helsinki, and approval was obtained from the Institutional Review Board of the Bursa Uludag University School of Medicine (2023-16/29). Patients subjected to off-label use of the implant were briefed accordingly. Following a comprehensive explanation of the procedure, written informed consent for the injection was obtained from each patient prior to administration.

The Shapiro–Wilk test was used to assess normality, with continuous variables expressed as median and range, and categorical variables presented as frequencies. Fisher’s exact test was employed to compare distributions of categorical variables. Repeated measures ANOVA and one-way ANOVA were utilised for comparative statistical analysis. All statistical tests were two-tailed, with a significance level set at 0.05. Statistical analyses were conducted using IBM SPSS Statistics version 28.0 (IBM Corp. Released 2021. IBM SPSS Statistics for Windows, Version 28.0. Armonk, NY: IBM Corp), and graphics were generated with GraphPad Prism version 8 (Free Trial, GraphPad Software, Inc.).

## Results

Thirty-nine eyes from 38 patients were included in this study. Nineteen eyes from 19 patients received SC-DEX injections, while 20 eyes from 19 patients received IV-DEX injections (Table 1). There was no significant difference between the two groups regarding demographics, indications for injections, or lens status. Ten patients in the SC-DEX group were phakic, compared to 8 patients in the IV-DEX group. The primary indications for the injections were diabetic macular oedema in 22 eyes, branch retinal vein occlusion in 4 eyes, central retinal vein occlusion in 3 eyes, uveitic macular oedema in 7 eyes, and postoperative cystoid macular oedema in 3 eyes (Table 1).

**Table 1:** Demographics and clinical characteristics of the patients in each group.

Groups	Supraciliary	Intravitreal
Age	58 (31–79)	62 (36-79)
Lens status		
Phakic	10	8
Pseudophakic	9	11
Sex		
F	10	13
M	9	6
Indications		
DMO	10	12
BRVO	1	3
CRVO	1	2
UCMO	5	2
PCMO	2	1
Number of patients with capsule-lens compromise	1 (SFIOL)	1 (laser capsulotomy)

*BRVO* branch retinal vein occlusion, *CRVO* central retinal vein occlusion, *DMO* diabetic macular oedema, *F* female, *M* male, *PCMO* pseudophakic cystoid macular oedema, *SFIOL* scleral fixated intraocular lens, *UCMO* uveitic cystoid macular oedema.

**Fig. 1:**
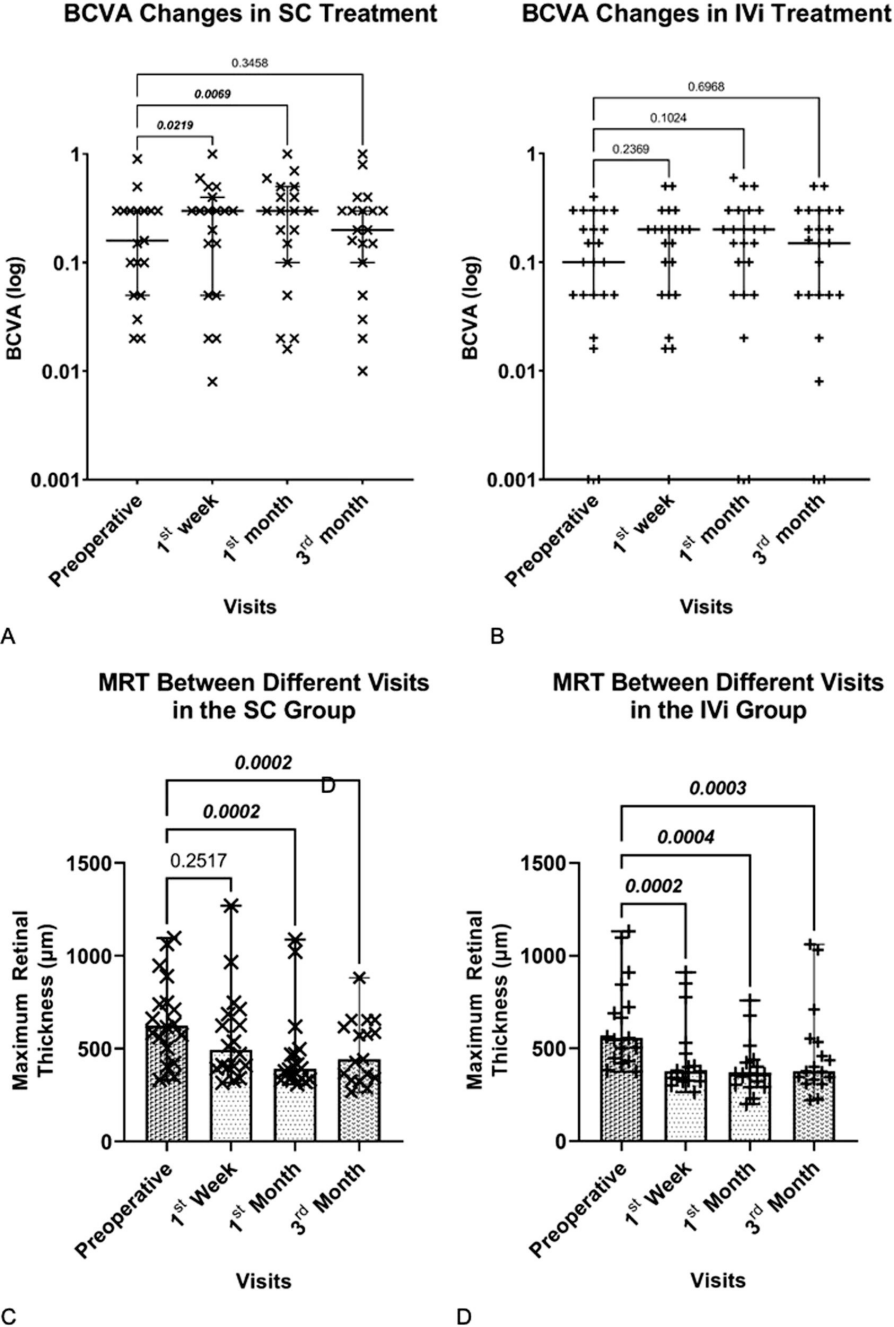
Maximum retinal thickness and logarithmic BCVA values of each study group in every visit. **A** Maximum retinal thickness of the IVi group. **B** Maximum retinal thickness of the SC group. **C** Logarithmic BCVA values of the IVi group. **D** Logarithmic BCVA values of the SC group. MRT maximum retinal thickness, BCVA best-corrected visual acuity, IVi intravitreal, SC supraciliary.

In this study, baseline BCVA levels had no significant difference between the 2 groups (*p* = 0.639). However, in the SC-DEX group, BCVA significantly improved compared to baseline levels at both the 1st week (*p* = 0.0219) and the 1st month (*p* = 0.0069). No significant difference in BCVA was observed at the 3rd month compared to baseline levels (*p* = 0.169). In contrast, the IV-DEX group did not exhibit significant differences in BCVA compared to baseline levels at the postoperative 1st week, 1st month, and 3rd month (*p* = 0.2369, *p* = 0.1024, and *p* = 0.6968, respectively) (Fig. 1A, B).

Overall, a significant difference in BCVA was observed between the two groups in the postoperative 1st week (*p* = 0.014), with the SC-DEX group showing superior median BCVA levels compared to the IV-DEX group. No significant differences in BCVA were noted between the postoperative 1st and 3rd months (*p* = 0.078 and *p* = 0.745, respectively).

There was no statistically significant difference between the two groups in terms of CRT at the 1st week, 1st month, and 3rd month visits (*p* values for the 1st week, 1st month, and 3rd month were *p* = 0.425, *p* = 0.052, and *p* = 0.625, respectively). Regarding ΔCRT, no significant differences were found between the groups at any visit, with *p* values at the 1st week, 1st month, and 3rd month being 0.17, 0.4075, and 0.9773, respectively (Table 2).

**Table 2:** Central retinal thickness (CRT) levels in study groups at different visits and differences in central retinal thickness (ΔCRT) between visits.

	CRT (in µm)		CRT Changes (ΔCRT, in µm)	
	IV-DEX	SC-DEX	IV-DEX	SC-DEX
Preoperative	479 (300–1020)	533 (258–909)	Baseline	
	*p* = 0.425		185 (6–541)	51 (−191–314)
Postoperative 1st week	300 (213–793)	402 (173–1100)	*p* = 0.17	
	*p* = 0.052		229 (5–707)	169.5 (−124–473)
Postoperative 1st month	286 (136–712)	319 (175–1033)	*p* = 0.4075	
	*p* = 0.516		150 (−43–484)	102.5 (−62–392)
Postoperative 3rd month	318 (167–967)	388.5 (155–768)	*p* = 0.9773	
	*p* = 0.625			

In the SC treatment group, MRT significantly decreased at the 1st and 3rd month visits compared to preoperative measurements (*p* = 0.0002 for both), but not at the 1st week visit (*p* = 0.2517). Significant reductions from baseline were noted at the 1st week, 1st month, and 3rd month in the IV-DEX group (*p* = 0.0002, *p* = 0.0004, and *p* = 0.0003, respectively) (Fig. 1C, D).

**Fig. 2:**
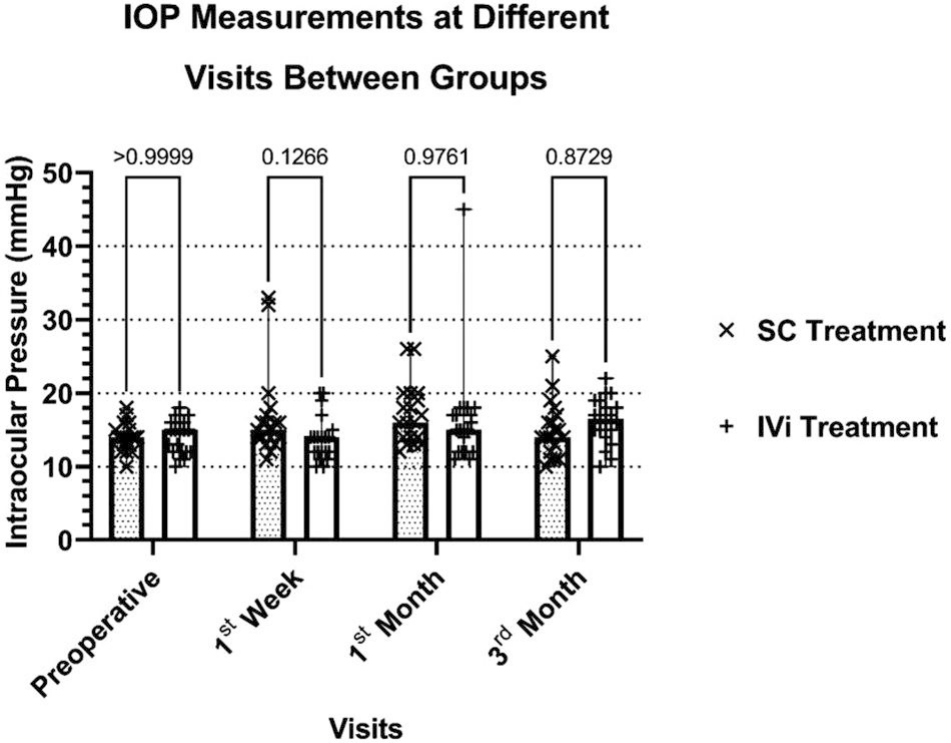
Different charts show the CRT and IOP changes among different visits. CRT central retinal thickness, IOP intraocular pressure.

In this study, we monitored IOP fluctuations, particularly in cases receiving SC-DEX injections. However, no significant differences were observed between the 2 groups in IOP measurements at baseline, and at the postoperative 1st week, 1st month, and 3rd month (*p* = 0.250, *p* = 0.078, *p* = 0.888, *p* = 0.260) (Fig. 2). No instances of ocular hypertony or hypotony were detected on the 1st postoperative day in patients who received SC-DEX or IV-DEX implants. In the SC treatment group, IOP increased to the mid-thirties in one patient. In the IV-DEX treatment group, IOP rose to the forties in one patient and to the mid-twenties in two patients, all of whom responded to topical antiglaucoma treatment.

**Fig. 3:**
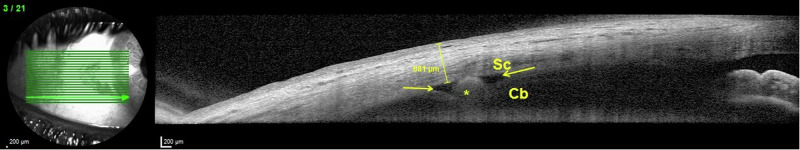
Anterior segment optical coherence tomography of a patient with suprachoroidal DEX implantation in the postoperative first week. The anterior segment optical coherence tomography shows the implant (yellow asterisks). The image demonstrates that the suprachoroidal space, previously a potential space, has become visible due to implantation, indicated by yellow arrows. Cb ciliary body, Sc sclera.

SC-DEX implants were visualised in all eyes after SC injections using anterior segment OCT (Fig. 3). Fortunately, no cases of suprachoroidal haemorrhage, choroidal detachment, ciliary body detachment, or endophthalmitis were observed in either group. Nine patients (47.3%) in the SC-DEX group reported pain during injections, and all eyes in this group developed subconjunctival haemorrhage. Among these eyes, 4 (21%) had haemorrhage in 1 quadrant, 13 (68.4%) had haemorrhage in 2 quadrants, and 2 (10.5%) had haemorrhage in 3 quadrants. The progression of cataract could not be assessed in either group due to the short follow-up of the study.

In the SC-DEX treatment group, 4 patients (21.05%) experienced clinically significant macular oedema recurrence before 3 months and required re-injections. In the IV-DEX treatment group, 2 cases (10%) required repeat treatment. Due to the preliminary nature of the study, all cases requiring re-injections were treated with additional IV-DEX injections. Follow-up of these repeat treatments showed that 5 patients (26.32%) in the SC-DEX group and 4 patients (20%) in the IV-DEX group required further IV-DEX treatments after 3 months. There were no statistically significant differences between the two treatment groups in the number of patients requiring treatment before or after 3 months (*p* = 0.318 and *p* = 0.832, respectively).

## Discussion

The 700-µg preservative-free sustained-release DEX implant is utilised as an intravitreal treatment to maintain therapeutic levels within the vitreous for an extended period [14]. At present, the DEX implant has emerged as a valuable option for managing posterior segment diseases. However, it carries certain pharmacological risks, including steroid-induced cataracts and glaucoma, as well as non-pharmacological complications such as anterior chamber migration, vitreous haemorrhage, and retinal detachment [15]. Essentially, the pharmacological risk is significantly lower compared to other intravitreal steroid injections [16]. Evidence indicates that intravitreal triamcinolone can increase IOP by approximately 35%, whereas the DEX implant increases IOP by about 15% in cases of retinal vein occlusion [17, 18]. Additionally, the relative risk of cataract progression with the DEX implant is about 3.5 times lower than with intravitreal triamcinolone [16].

However, this favourable pharmacological safety profile may be compromised by non-pharmacological adverse events that can occur when a cylindrical implant, measuring 6.0 mm in length and 0.46 mm in diameter, is injected through the pars plana using a 22-gauge needle. The mechanical trauma at the injection site, along with the proximity of the sharp needle to the retina, could potentially cause vitreous haemorrhage, retinal detachment, or the introduction of microorganisms into the eye. Moreover, in cases with previous history of vitrectomy, defective iris tissue, zonulo-capsular diaphragm instability, or anterior hyaloid face rupture, the DEX implant may migrate into the anterior chamber, potentially leading to irreversible corneal decompensation [14, 19, 20]. While ongoing efforts aim to enhance the safety of DEX implantation into the vitreous, concurrent evaluations are exploring new strategies to reduce the side effect profile while maintaining the efficacy of the DEX implant [21, 22].

Approval of suprachoroidal injection of TA by FDA has represented a breakthrough in this field. Previous studies have demonstrated that the concentration of TA in the posterior segment is 12 times higher with suprachoroidal injection compared to intravitreal injection. With only 3% of the drug entering to the anterior chamber [10, 11], steroid exposure within the anterior segment is minimised and the risk of IOP elevation and cataract formation is reduced with suprachoroidal injections [23].

The results in our study showed that anterior segment OCT can be used as a tool to monitor the placement of the SC implant. Here in our cases, SC-DEX treatment was well tolerated and anatomical and visual efficacy and safety parameters were comparable to that of IV-DEX treatment. BCVA levels were significantly better in the SC-DEX group compared to the IV-DEX group during the first week of injections (*p* = 0.014). When considering both groups, although baseline BCVA was not statistically significant, the lower BCVA in the IV-DEX group may have contributed to the limited improvement after the IV-DEX injection, which differed from the literature [24]. No differences were observed between the two groups at any visit in terms of IOP fluctuations. However, due to the short follow-up period of the study, cataract progression could not be assessed in either group.

Our findings showed that the reduction of MRT was more pronounced in the 1st and 3rd months of follow-up compared to the 1st week. However, the duration of efficacy was relatively shorter in the SC-DEX group than the IV-DEX group. Although not statistically significant, the incidence of retreatments was slightly higher in the SC-DEX group. These differences may be attributed to pharmacokinetics within the SC space. Initially, the implant may exhibit a slow-release profile in the SC space, while the increased clearance of aqueous-soluble substances over time could contribute to early depletion [25–28]. The reduced effectiveness of the DEX implant in vitrectomized eyes may serve as a reference for demonstrating this mechanism [29]. In terms of MRT, a statistically significant difference was observed in the SC-DEX group in the postoperative period across different visits, except for the first week (Fig. 1C, D). Regarding CRT values, there was no statistically significant difference between the two groups at any visit, nor was there a statistically significant difference in CRT changes between groups (Table 2). However, the lack of significance in MRT change at the first week and the near-significant difference in CRT values during the first week may suggest a slower onset of the SC-DEX implant’s effect. Hydrophilic drugs are cleared via choroidal circulation, which may explain the weaker initial effect due to the early clearance of dexamethasone from the DEX implant. However, the relatively small sample size may also account for this finding. Since suprachoroidal DEX implant administration is novel in the literature, further studies are required to evaluate this observation.

Despite these promising early results, SC-DEX therapy may still raise concerns about its safety profile. Fortunately, no vision threatening complications occurred in either treatment groups. Along with further studies on safety, adhering to proper patient selection and injection technique could help prevent undesirable consequences [25, 29]. Previous studies on suprachoroidal application of corticosteroids have demonstrated optimistic outcomes. In a phase 3 study of suprachoroidal TA for noninfectious uveitis, the rate of increased IOP was 11.5%, which is lower than the 25–43% incidence reported with intravitreal DEX implants for uveitic macular oedema [8]. In an animal study by Chen et al., the authors suggested that the increase of IOP in suprachoroidal TA could be volume-dependent [12].

It is known that IV-DEX implantation can lead to cataract formation, with the risk increasing with repeated injections [14]. Reports indicate that 12.4% of eyes treated with IV-DEX implants required cataract surgery during follow-up [30]. Additionally, rapidly progressing cataracts have been reported after IV-DEX injections, potentially due to trauma from the injection procedure or contact with the intravitreal implant [31]. SC-DEX injection offers the advantage of preventing some non-pharmacological complications such as capsular damage to the lens and the risk of implant migration into the anterior chamber.

The results obtained in this study are important as they demonstrate that the SC space is amenable to injections beyond those previously established. To our knowledge this is the first study to investigate the efficacy and safety of SC-DEX injections. One of the strengths of the study is its comparative evaluation of outcomes for both intravitreal and SC injections. However, this study has several limitations. These include its preliminary nature, the relatively small sample size, off-label use of the implant, and the lack of multicentre collaboration. Additionally, the effect on macular oedema due to different indications, its impact on vitrectomized eyes, and its effects on naïve eyes should also be investigated, similar to intravitreal DEX implants [32, 33].

Given that various biopolymers are biocompatible, effective, and capable of extending the half-life of drugs in the suprachoroidal space, there is considerable potential for emerging therapies utilising various drugs administered via the suprachoroidal space [25]. Currently, the dexamethasone implant stands out as a leading candidate for this application.

In this context, detailed safety assessments conducted using the SC- DEX application can enable a thorough review and refinement of existing standard treatment approaches. This process may particularly support the development of personalised treatment strategies for cases with intravitreal related risks and facilitate modifications to treatment protocols. Specialised designs and materials may be necessary to facilitate injection into this area and ensure compatibility with the surrounding tissue.

In conclusion, this preliminary study suggests that SC injection of the DEX implant is a promising alternative for managing posterior segment diseases. It demonstrates effectiveness comparable to intravitreal administration, with the potential for similar or reduced complications. Comparative, multicentre studies with large sample sizes and the use of multimodal imaging are needed to confirm the efficacy and safety of SC-DEX injections and beyond.

## Summary

### What was known before

- It was known that corticosteroids are potent anti-inflammatory drugs widely used in ophthalmology for both anterior and posterior segment diseases due to their anti-inflammatory and anti-neovascularisation properties.
- Additionally, suprachoroidal and intravitreal steroid applications were recognised for their effectiveness in bypassing ocular barriers, resulting in fewer systemic and local side effects compared to other routes.

### What this study adds

- Findings suggest the supraciliary dexamethasone implant is as effective as the intravitreal, with potentially fewer complications and better initial visual improvements, indicating its viability as an alternative treatment.

## Supplementary information

Supplementary Material 1.

## References

[1] Cunningham MA, Edelman JL, Kaushal S. Intravitreal steroids for macular edema: the past, the present, and the future. Surv Ophthalmol. 2008;53:139–49. 18348879 10.1016/j.survophthal.2007.12.005

[2] Fung AT, Tran T, Lim LL, Samarawickrama C, Arnold J, Gillies M, et al. Local delivery of corticosteroids in clinical ophthalmology: a review. Clin Exp Ophthalmol. 2020;48:366–401. 31860766 10.1111/ceo.13702 PMC7187156

[3] Thorne JE, Sugar EA, Holbrook JT, Burke AE, Altaweel MM, Vitale AT, et al. Periocular triamcinolone vs. intravitreal triamcinolone vs. intravitreal dexamethasone implant for the treatment of uveitic macular edema: the PeriOcular vs. INTravitreal corticosteroids for uveitic macular edema (POINT) Trial. Ophthalmology. 2019;126:283–95. 30269924 10.1016/j.ophtha.2018.08.021 PMC6348060

[4] Zur D, Iglicki M, Loewenstein A. The role of steroids in the management of diabetic macular edema. Ophthalmic Res. 2019;62:231–6. 31048580 10.1159/000499540

[5] Emi K, Pederson JE, Toris CB. Hydrostatic pressure of the suprachoroidal space. Invest Ophthalmol Vis Sci. 1989;30:233–8. 2914753

[6] Jose-Vieira R, Ferreira A, Meneres P, Sousa-Pinto B, Figueira L. Efficacy and safety of intravitreal and periocular injection of corticosteroids in noninfectious uveitis: a systematic review. Surv Ophthalmol. 2022;67:991–1013. 34896190 10.1016/j.survophthal.2021.12.002

[7] Thomas J, Kim L, Albini T, Yeh S. Triamcinolone acetonide injectable suspension for suprachoroidal use in the treatment of macular edema associated with uveitis. Expert Rev Ophthalmol. 2022;17:165–73. 36060305 10.1080/17469899.2022.2114456 PMC9438525

[8] Yeh S, Henry CR, Kapik B, Ciulla TA. Triamcinolone acetonide suprachoroidal injectable suspension for uveitic macular edema: integrated analysis of two phase 3 studies. Ophthalmol Ther. 2023;12:577–91. 36399237 10.1007/s40123-022-00603-x PMC9834475

[9] Nawar AE. Effectiveness of suprachoroidal injection of triamcinolone acetonide in resistant diabetic macular edema using a modified microneedle. Clin Ophthalmol. 2022;16:3821–31. 36438589 10.2147/OPTH.S391319 PMC9698330

[10] Goldstein DA, Do D, Noronha G, Kissner JM, Srivastava SK, Nguyen QD. Suprachoroidal corticosteroid administration: a novel route for local treatment of noninfectious uveitis. Transl Vis Sci Technol. 2016;5:14. 27980877 10.1167/tvst.5.6.14 PMC5156441

[11] Marashi A, Baba M, Abu Ghedda S, Kitaz MN, Zazo A. A combination of suprachoroidal injection of triamcinolone using a custom-made needle and intravitreal Ziv-aflibercept every eight weeks to manage naive/denovo central DME: a single-center retrospective case series. Int J Retina Vitreous. 2024;10:30. 38566193 10.1186/s40942-024-00550-8 PMC10986050

[12] Chen M, Li X, Liu J, Han Y, Cheng L. Safety and pharmacodynamics of suprachoroidal injection of triamcinolone acetonide as a controlled ocular drug release model. J Control Release. 2015;203:109–17. 25700623 10.1016/j.jconrel.2015.02.021

[13] Ciulla TY S. Microinjection via the suprachoroidal space: a review of a novel mode of administration. Am J Manag Care. 2022;28:S243–S52. 36395492 10.37765/ajmc.2022.89270

[14] Iovino C, Mastropasqua R, Lupidi M, Bacherini D, Pellegrini M, Bernabei F, et al. Intravitreal dexamethasone implant as a sustained release drug delivery device for the treatment of ocular diseases: a comprehensive review of the literature. Pharmaceutics. 26;12:703. 10.3390/pharmaceutics12080703 PMC7466091 32722556

[15] Boyer DS, Yoon YH, Belfort R Jr, Bandello F, Maturi RK, Augustin AJ, et al. Three-year, randomized, sham-controlled trial of dexamethasone intravitreal implant in patients with diabetic macular edema. Ophthalmology. 2014;121:1904–14. 24907062 10.1016/j.ophtha.2014.04.024

[16] Mishra SK, Gupta A, Patyal S, Kumar S, Raji K, Singh A, et al. Intravitreal dexamethasone implant versus triamcinolone acetonide for macular oedema of central retinal vein occlusion: quantifying efficacy and safety. Int J Retina Vitreous. 2018;4:13. 29632703 10.1186/s40942-018-0114-2 PMC5883339

[17] Haller JA, Bandello F, Belfort R Jr, Blumenkranz MS, Gillies M, Heier J, et al. Dexamethasone intravitreal implant in patients with macular edema related to branch or central retinal vein occlusion twelve-month study results. Ophthalmology. 2011;118:2453–60. 21764136 10.1016/j.ophtha.2011.05.014

[18] Ip MS, Scott IU, VanVeldhuisen PC, Oden NL, Blodi BA, Fisher M, et al. A randomized trial comparing the efficacy and safety of intravitreal triamcinolone with observation to treat vision loss associated with macular edema secondary to central retinal vein occlusion: the Standard Care vs Corticosteroid for Retinal Vein Occlusion (SCORE) study report 5. Arch Ophthalmol. 2009;127:1101–14. 19752419 10.1001/archophthalmol.2009.234 PMC2872173

[19] Rock D, Bartz-Schmidt KU, Rock T. Risk factors for and management of anterior chamber intravitreal dexamethasone implant migration. BMC Ophthalmol. 2019;19:120. 31138164 10.1186/s12886-019-1122-1 PMC6537356

[20] Kiristioglu MO, Gunduz GU, Abdullayeva N, Doganay S, Yalcinbayir O. Non-pharmacological aspects of intravitreal dexamethasone implant injections: a retrospective study of 3430 injections and complications. Retina. 2024;45:95-106. 10.1097/IAE.0000000000004270 39312882

[21] Chin EK, Almeida DRP, Velez G, Xu K, Peraire M, Corbella M, et al. Ocular hypertension after intravitreal dexamethasone (ozurdex) sustained-release implant. Retina. 2017;37:1345–51. 27806001 10.1097/IAE.0000000000001364 PMC5411345

[22] Mateo C, Alkabes M, Bures-Jelstrup A. Scleral fixation of dexamethasone intravitreal implant (OZURDEX(R)) in a case of angle-supported lens implantation. Int Ophthalmol. 2014;34:661–5. 23928945 10.1007/s10792-013-9841-4

[23] Khor HG, Lott PW, Wan Ab Kadir AJ, Singh S, Iqbal T. Review of risk factors and complications of anterior migration of ozurdex implant: lessons learnt from the previous reports. J Ocul Pharm Ther. 2024;40:342–60. 10.1089/jop.2023.0012 37676992

[24] Mello Filho P, Andrade G, Maia A, Maia M, Biccas Neto L, Muralha Neto A, et al. Effectiveness and safety of intravitreal dexamethasone implant (ozurdex) in patients with diabetic macular edema: a real-world experience. Ophthalmologica. 2019;241:9–16. 30408801 10.1159/000492132

[25] Naftali Ben Haim L, Moisseiev E. Drug delivery via the suprachoroidal space for the treatment of retinal diseases. Pharmaceutics. 2021;13:967. 10.3390/pharmaceutics13070967 PMC8309112 34206925

[26] Wang M, Liu W, Lu Q, Zeng H, Liu S, Yue Y, et al. Pharmacokinetic comparison of ketorolac after intracameral, intravitreal, and suprachoroidal administration in rabbits. Retina. 2012;32:2158–64. 23099451 10.1097/IAE.0b013e3182576d1d

[27] Olsen TW, Feng X, Wabner K, Csaky K, Pambuccian S, Cameron JD. Pharmacokinetics of pars plana intravitreal injections versus microcannula suprachoroidal injections of bevacizumab in a porcine model. Invest Ophthalmol Vis Sci. 2011;52:4749–56. 21447680 10.1167/iovs.10-6291 PMC3175963

[28] Abarca EM, Salmon JH, Gilger BC. Effect of choroidal perfusion on ocular tissue distribution after intravitreal or suprachoroidal injection in an arterially perfused ex vivo pig eye model. J Ocul Pharm Ther. 2013;29:715–22. 10.1089/jop.2013.0063 23822159

[29] Patel SR, Lin AS, Edelhauser HF, Prausnitz MR. Suprachoroidal drug delivery to the back of the eye using hollow microneedles. Pharm Res. 2011;28:166–76. 20857178 10.1007/s11095-010-0271-y PMC3038673

[30] Rosenblatt A, Udaondo P, Cunha-Vaz J, Sivaprasad S, Bandello F, Lanzetta P, et al. A collaborative retrospective study on the efficacy and safety of intravitreal dexamethasone implant (ozurdex) in patients with diabetic macular edema: the European DME Registry Study. Ophthalmology. 2020;127:377–93. 31932090 10.1016/j.ophtha.2019.10.005

[31] Lee JH, Park JY, Kim JS, Hwang JH. Rapid progression of cataract to mature stage after intravitreal dexamethasone implant injection: a case report. BMC Ophthalmol. 2019;19:1. 30606142 10.1186/s12886-018-1008-7 PMC6318997

[32] Iglicki M, Busch C, Lanzetta P, Sarao V, Veritti D, Rassu N, et al. Vitrectomized vs non-vitrectomized eyes in DEX implant treatment for DMO-Is there any difference? the VITDEX study. Eye. 2023;37:280–4. 35043004 10.1038/s41433-022-01931-9 PMC9873723

[33] Iglicki M, Busch C, Zur D, Okada M, Mariussi M, Chhablani JK, et al. Dexamethasone implant for diabetic macular edema in naive compared with refractory eyes: the International Retina Group Real-Life 24-Month Multicenter Study. The IRGREL-DEX Study. Retina. 2019;39:44–51. 29697589 10.1097/IAE.0000000000002196

